# Efficacy of sub-threshold focused ultrasound irradiation against pancreatic cancer xenografts evaluated using magnetic resonance imaging

**DOI:** 10.18632/oncotarget.19241

**Published:** 2017-07-14

**Authors:** Rui Wang, Qian Guo, Yini Chen, Yihui Gao, Lei Wu, Bing Hu, Lixin Jiang

**Affiliations:** ^1^ Department of Ultrasonography, Shanghai Jiaotong University Affiliated No. 6 Hospital, Shanghai 200233, PR China; ^2^ Shanghai Institute of Ultrasound in Medicine, Shanghai 200233, PR China

**Keywords:** pancreatic cancer, MRI, nude mice, focused ultrasound ablation, xenografts

## Abstract

We investigated the efficacy and optimal period for using magnetic resonance imaging (MRI) to detect effects of sub-threshold focused ultrasound (FUS) irradiation. Nude mice bearing pancreatic cancer xenografts were subjected to MRI and pathology examnation before, and 24 h, 48 h, 2 weeks after irradiation, which were used to evaluate therapeutic effects of FUS. Tumor volumes were lower post-treatment than control group (P < 0.05). The T1WI turbo spin echo (T1WI-TSE) sequence was similar signal before and after treatment. On T1 enhanced scanning sequence (T1WI-SPIR) imaging, ablation lesions appeared as patchy areas of low signal after 24 h and 48 h. After 2 weeks, the ablation lesions contained low signal areas with clear borders. Hematoxylin and eosin (HE) staining revealed small vessels at ablation lesions with no obvious boundary between cell injury areas and normal tumor cells areas in early-stage, while revealed obvious boundaries 2 weeks post-treatment. Terminal deoxynucleotidyl transferase-modified, dUTP nick-end labeling (TUNEL) staining showed cell apoptosis in early-stage, and revealed reduced apoptotic cells and increased necrotic cell areas 2 weeks later. These findings indicate sub-threshold FUS induces pancreatic cancer cell apoptosis and inhibits tumor growth. Contrast-enhanced MRI delineated the ablation lesions better 2 weeks post-treatment than early stage.

## INTRODUCTION

High-intensity focused ultrasound (HIFU) is a minimally invasive technology for cancer treatment. It uses a FUS transducer to concentrate low-energy ultrasound waves and increase the temperature above 60°C in tumor tissue causing coagulative necrosis [1]. HIFU is used to treat benign and malignant solid tumors such as uterine fibroids, pancreatic cancer, hepatic carcinoma, breast cancer, and osteosarcoma [2–6]. Focused ultrasound (FUS) has demonstrated efficacy in the treatment of pancreatic cancer [7, 8]. As a retroperitoneal organ, the pancreas is surrounded anteriorly by the gastrointestinal tract, blood vessels, and biliary tract. Pancreatic tissues contain large amounts of digestive enzymes. Animal experiments and clinical studies have confirmed that improper doses of FUS therapy can result in severe side effects such as damage to normal tissues outside the target region [9, 10]. Reduction of the FUS dose can prevent these adverse effects.

Magnetic resonance imaging (MRI) and contrast-enhanced MRI (CE-MRI) play important roles in FUS therapy efficacy evaluation and follow-up. These methods can detect tumor and ablation lesion morphology (e.g. size and contours), and perfusion defects within ablation regions. An increase in tissue injury after local heat therapy has been associated with the progression of microvascular injury [11, 12]. The effects of FUS on microvessels may extend beyond the target regions [13, 14]. Reduced FUS doses can induce apoptosis and reduce coagulation necrosis. We investigated the efficacy and the optimal period for using magnetic resonance imaging (MRI) to detect sub-threshold FUS. We compared the results with pathological data to determine whether CE-MRI was feasible for the evaluation of the early efficacy of FUS.

## RESULTS

**Figure 1:**
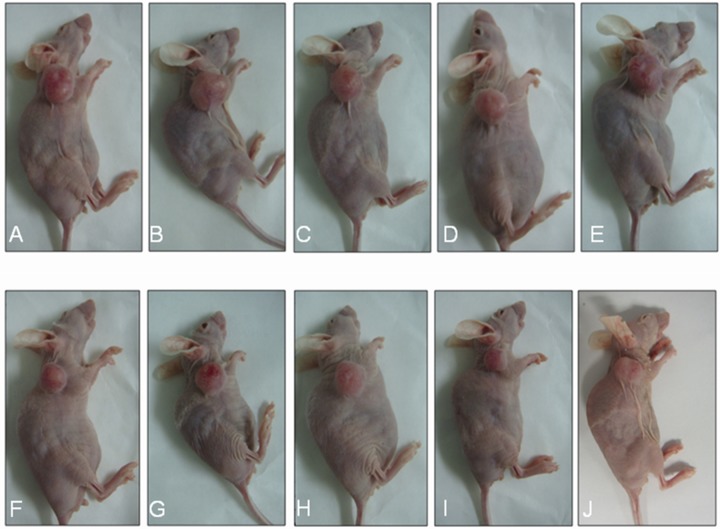
Representative xenograft tumors in the control and HIFU treatment groups at various times after therapy **(A-E)** Representative tumors in the control group at 0, 3, 6, 9, and 15 days after treatment. **(F-J)** Representative tumors in the HIFU group at 0, 3, 6, 9, and 15 days after treatment.

The average ± standard deviation (SD) tumor volume before treatment was 538.98 ± 15.04 mm^3^ and 548.72 ± 27.94 mm^3^ in the control and HIFU treatment groups (P > 0.05), respectively. Representative tumors in the control and HIFU treatment groups 0, 3, 6, 9, and 15 days after irradiation are shown in Figure 1. The average ± SD tumor volume 15 days after irradiation was 1,746.58 ± 312.77 mm^3^ and 1,085.23 ± 217.13 mm^3^ in the control and HIFU treatment groups (P < 0.05), respectively. Tumor volumes in the control and FUS groups 0, 3, 6, 9, and 15 days after irradiation are shown in Table 1. The average tumor volume was larger in the control than HIFU treatment group. Xenograft tumor nodules exhibited irregular shapes. The skin surrounding the tumor was thinner and brighter and contained more nascent vessels. Tumor volumes were smaller in the HIFU group and there were fewer nascent vessels observed.

**Table 1:** Differences in tumor volume at various times between the control and FUS treatment groups (average ± SD: mm^3^)

Group	Before	3 days	6 days	9 days	12 days	15 days
Control	538.98 ± 15.03	625.44 ± 28.11	803.84 ± 40.20	977.11 ± 39.58	1288.96 ± 132.31	1746.58 ± 312.77
FUS	548.72 ± 27.94	599.26 ± 35.71	675.10 ± 61.00	790.55 ± 84.44	909.28 ± 127.26	1085.23 ± 217.13

### MRI of pancreatic cancer xenografts

**Figure 2:**
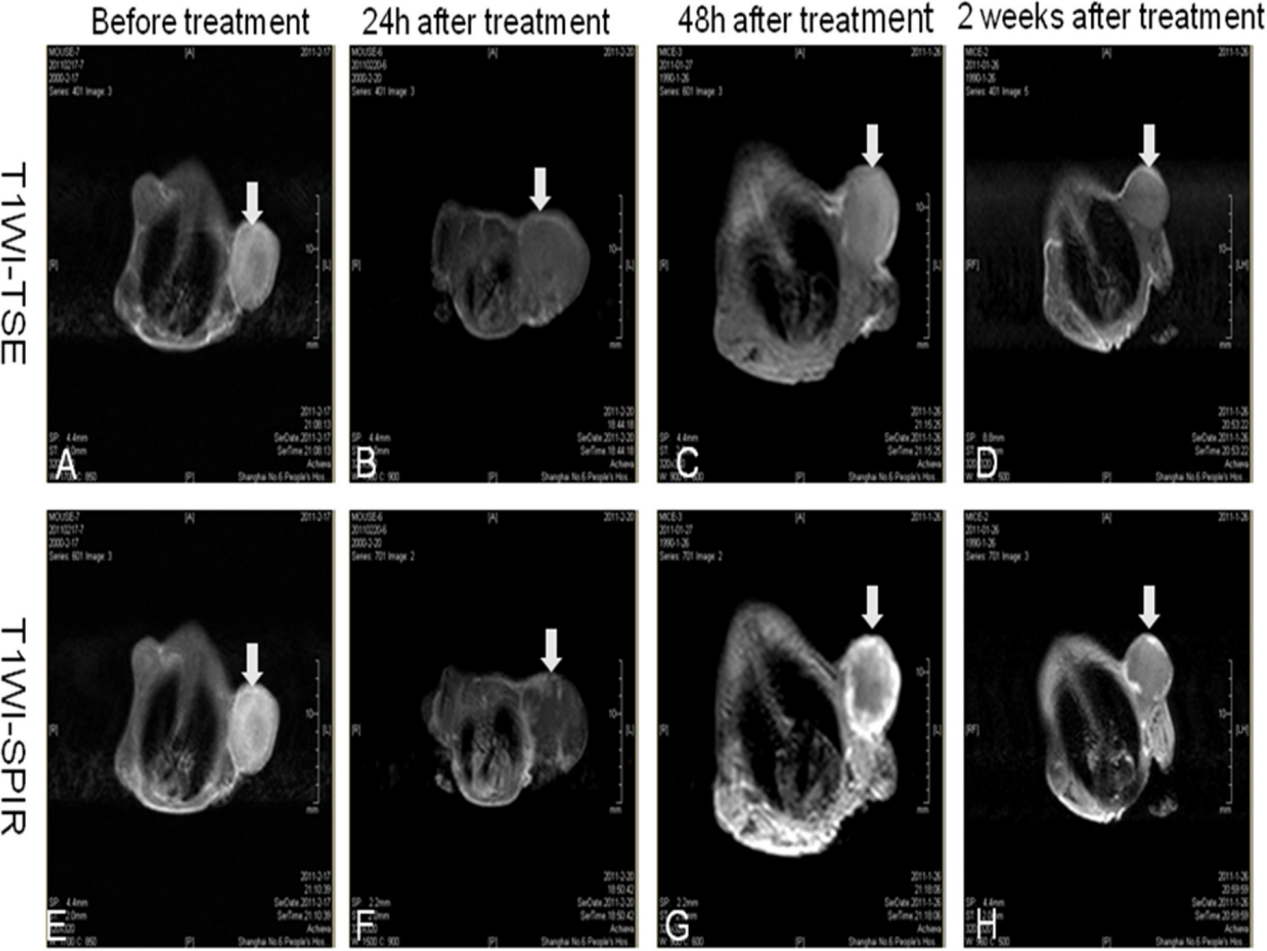
MRI of xenograft tumors at various times after therapy **(A-D)** Images from the T1WI-TSE sequence performed on subcutaneous pancreatic cancer xenografts in nude mice before FUS therapy and 24 h, 48 h, 2 weeks after HIFU therapy. **(E-H)** Images from the T1WI-SPIR sequence performed on xenografts enhancement before FUS therapy, and 24 h, 48 h, 2 weeks after HIFU therapy.

The T1WI-TSE sequence of subcutaneous pancreatic cancer xenografts in nude mice before FUS therapy displayed either a homogeneously low signal, iso-signal, or a mix of low and high signals (Figure 2A). The T1WI-SPIR sequence images of xenografts enhancement were divided into two types: those with strong signals and heterogeneous internal enhancement, and those with weak signals and enhancement at the edge of the tumors (Figure 2E).

### MRI of pancreatic cancer xenograft tumors at various time points after FUS therapy

Twenty-four hours after FUS therapy, the T1WI-TSE sequence showed mixed low and high signal areas (Figure 2B). On T1WI-SPIR sequence imaging, the ablation lesion demonstrated patchy, irregular, and low signal areas. Flocculent and cord-like enhancement areas were observed at the edge of the tumor (Figure 2F).

Forty-eight hours after FUS therapy, xenograft tumors showed iso-signal or high signal areas on T1WI-TSE sequences (Figure 2C). For the T1WI-SPIR sequence, the ablation lesions demonstrated irregular and non-enhanced low signal areas within tumor foci. Cord-like enhanced signals were observed at the edges of tumor foci (Figure 2G).

Two weeks after FUS therapy, the T1WI-TSE sequence demonstrated iso-signal or high signal areas (Figure 2D). The T1WI-SPIR sequence demonstrated that ablation lesions in the tumors had non-enhanced low signal areas with clear borders. Contrast-enhanced MRI delineated the ablation lesions better 2 weeks post-treatment than in the early phase after treatment (Figure 2H).

### Pathological evaluation of xenografts

**Figure 3:**
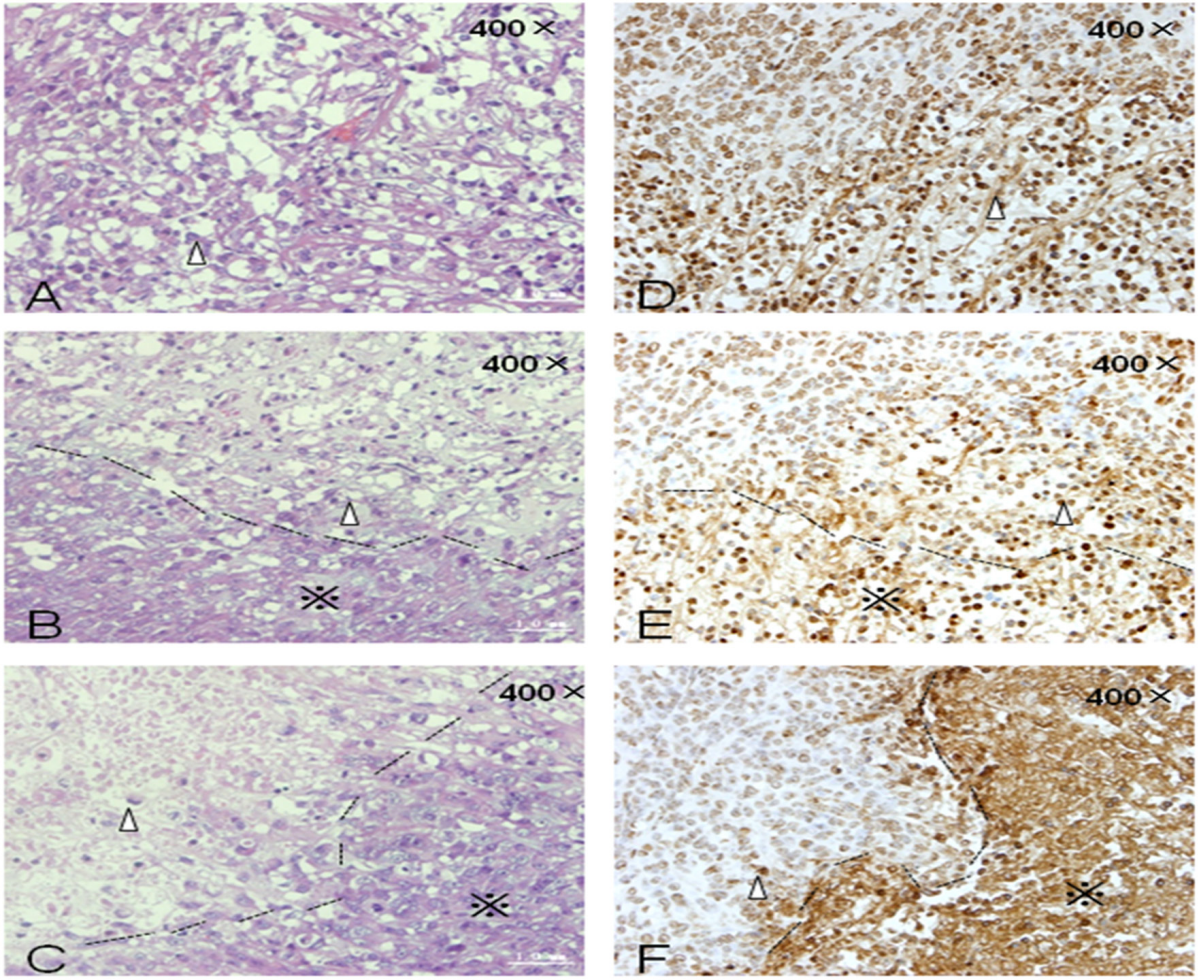
HE and TUNEL staining of xenograft tumors at various times after FUS treatment **(A)** HE staining 24 h after treatment. **(B)** HE staining 48 h after treatment. **(C)** HE staining 2 weeks after treatment. **(D)** TUNEL staining 24 h after treatment. **(E)** TUNEL staining 48 h after treatment. **(F)** TUNEL staining 2 weeks after treatment. Δ: Cells in ablation lesions underwent apoptosis and necrosis. Dotted line: Boundary between areas of cell injury and surrounding normal tumor cells. (※): Surrounding normal tumor cells.

HE staining revealed apoptotic and necrotic cells in ablation lesions 24 h after FUS therapy. No obvious boundary was observed between the areas of cell injury and surrounding normal tissue (Figure 3A). Small vessels were observed within the ablation lesions due to congestion and expansion of microvessels in early-stage, FUS-treated xenografts. Forty-eight hours after FUS therapy, the boundaries of ablation lesions were better delineated, the distribution of small vessels in ablation lesions decreased, and cellular structures appeared degraded. Large patches of necrotic and apoptotic cells were observed in tissues within the target areas (Figure 3B). Two weeks after FUS therapy, tissues in the ablation areas showed homogeneous structures without cells. The organization of the structures was better visualized due to tissue necrosis and cellulose formation in the ablation lesions. Tissue dissolution and absorption occurred between necrotic areas and small lacunae were visible (Figure 3C).

Terminal deoxynucleotidyl transferase-modified, dUTP nick-end labeling (TUNEL) staining 24 h after FUS therapy revealed apoptotic cells within the ablation lesions. Brown staining of the nuclei of apoptotic cells indicative of DNA fragmentation was observed (Figure 3D) along with chromatin margination and apoptotic bodies. Many apoptotic and necrotic cells were still detected within the ablation lesions 48 h after FUS therapy (Figure 3E). The number of apoptotic and necrotic cells decreased 2 weeks after treatment. Nuclear condensation and brown-colored staining were also observed (Figure 3F).

## DISCUSSION

Accurate and objective evaluation of changes in lesions after FUS treatment is important for ensuring efficacy. MRI can detect changes in temperature within tissue with an accuracy of 1°C. During FUS therapy, observation of the areas heated by the focused beam using the temperature-sensitive T1WI-TSE sequence had an accuracy within 1 mm. These features can facilitate monitoring and localization by MRI during FUS treatment [15, 16]. MRI produces images with strong soft tissue resolution. MRI is also capable of multi-faceted and multi-sequence imaging. Therefore, it is advantageous for post-operative evaluation of FUS therapy. Given the pathological changes that occur after FUS therapy, evaluation of FUS efficacy using MRI and CE-MRI is better at approximately 1 week compared to immediately after treatment. Early evaluation may cause inaccurate determination of the treatment dose, leading to ineffective treatment.

Apoptosis was observed immediately following irradiation at the sub-threshold ablation temperature. The signal changes observed by MRI using the T1WI sequence were not significant compared to the signals before treatment. By using gadolinium chelates, CE-MRI can increase the imaging contrast between the untreated normal and tumor tissue and ablation lesions. Xenograft tumors demonstrated enhancement on CE-MRI before treatment. After treatment, these areas no longer demonstrated enhancement. The efficacy of FUS at the sub-threshold ablation temperature was evaluated. However, because enhancement of some xenografts is not obvious before treatment, efficacy evaluation after treatment is more difficult.

During the early phase after FUS therapy at the sub-threshold ablation temperature, CE-MRI demonstrated heterogeneous partial perfusion areas. The biological effects of FUS therapy at the sub-threshold ablation temperature were not sufficient to cause instant tissue necrosis. Tumor growth was inhibited through the induction of apoptosis. Therefore, efficacy evaluation immediately after FUS therapy may not be accurate due to possible contrast agent enhanced imaging in treatment areas. This may result in overtreatment. However, with a longer time interval after FUS therapy, ablation lesions gradually became fibrosed, absorbed, and exhibited clear boundaries. CE-MRI could better display the boundary and range of ablation lesions 2 weeks after treatment. In comparison, contrast-enhanced ultrasound may be able to evaluate the efficacy at an early stage after FUS therapy due to changes in the blood supply in the FUS target regions. In the future, we will evaluate the ability of contrast-enhanced ultrasound to assess the efficacy of FUS therapy during the early phase after treatment.

## MATERIALS AND METHODS

### Subjects

We established a subcutaneous xenograft mouse model using the PaTu 8988t human pancreatic cancer cell line derived from a liver metastasis of a primary pancreatic adenocarcinoma. This cell line was a gift from Professor Xingpeng Wang at the Shanghai First People's Hospital. PaTu 8988t cells were cultured in RPMI-1640 medium supplemented with 10% fetal bovine serum, 100 U/mL penicillin, and 100 U/mL streptomycin and maintained in a 37°C incubator with 5% CO_2_. Adherent cells were passaged every other day.

Twenty female BALB/c nude mice were obtained from the Chinese Academy of Sciences Shanghai Experimental Animal Center. The mice were 4–6 weeks of age and, weighed 20 ± 2.0 g. Mice were bred in SPF clean rooms at the Shanghai Sixth People's Hospital Experimental Animal Center. PaTu 8988t cells were diluted in RPMI-1640 medium and a single-cell suspension generated. The rate of living cells was > 95%. Following centrifugation, the supernatant was discarded, and the cells washed twice with PBS and resuspended in normal saline. A total of 1 × 10^7^ PaTu 8988t cells in a 0.2 mL suspension were subcutaneously injected into the right shoulders of nude mice. Tumor size was measured every 3 days using vernier calipers. The longest diameter (L) and width (W) were recorded. Tumor volume (V) was calculated using the following equation: V = 1/2 (L × W^2^).

### Experimental instruments

FUS treatment was performed with a HY2900 HIFU therapy system (Wuxi Haiying Techonology, Wuxi, China). The device consisted of an ultrasonic diagnostic unit under the control of a central processing unit. The 1.5 MHz therapeutic transducer (25 cm diameter, 14 cm focal length) consisted of six spherically focused elements. It was fixed on the top of a capsule filled with degassed water. The transducer was fixed on a computer-controlled, three-dimensional positioning device. Field mapping conducted at Wuxi Haiying Technology yielded an ellipsoidal focal volume with dimensions of 1.15 mm × 1.21 mm × 8.0 mm (−6 dB beam profile) based on the measurement of the field using a Tun001 hydrophone (NTR System, Inc., Onda Corp. Sunnyvale, CA, USA). Measurements were obtained at 0.64% of the maximum amplitude. Waveforms were recorded on a TDS5052 oscilloscope (Tektronix, Inc., Beaverton, OR, USA). The maximum electrical power from the amplifier to the therapy transducers was 1.02 kW. The corresponding total acoustic power of 479.2 W was measured using a Sartorius BS300 Balance (Beijing Sartorius Balance Co., Ltd, Beijing China). The spatially averaged intensity level at -6 dB was 11,340 W/cm^2^. All intensities shown were obtained in water.

A 3.5 MHz diagnostic transducer was positioned in the center of the therapeutic transducer to guide and monitor HIFU treatment. Tissues in the path of therapeutic ultrasound waves were visualized in diagnostic ultrasound images. The water bag had an acoustic transparent membrane bottom for HIFU to transmit without obstruction. The thermal needle (200 mm length, 1 mm outer diameter) was obtained from Haiying Electronic Medical System Co. (Wuxi, Jiangsu). The temperature-sensing point was located at the tip of the needle. MRI was performed with a Philips Achieva 3.0 T super-conductive magnetic resonance scanner (Eindhoven, Netherlands) combined with a four-channel imaging coil.

### Treatment of xenograft tumors in nude mice using sub-threshold FUS

**Figure 4:**
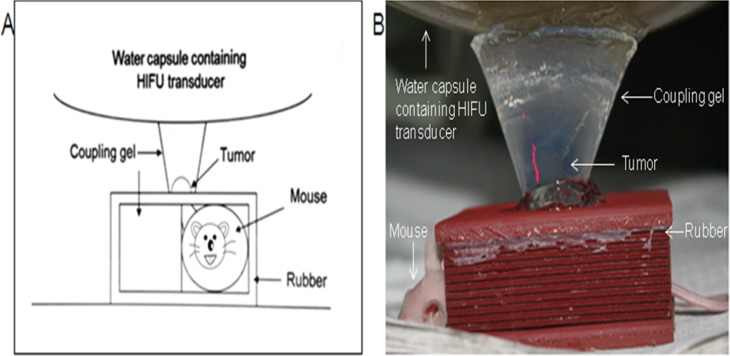
**(A)** Schematic of the experimental device. **(B)** Experimental device.

Ten tumor-bearing nude mice were anesthetized once the tumor volume reached approximately 500 mm^3^ with intraperitoneally injected pentobarbital sodium (50 mg/kg). Small animal protection molds were developed in-house using sound-absorbing rubber plates. Tumors could be accessed by the FUS device from the approximately 1.2 cm round hole in the mold. The top contact surfaces were coupled with ultrasonic coupling agents. The equipment is shown in Figure 4.

The highest rate of FUS-induced pancreatic cancer cell apoptosis occurred at the dose that produced the sub-threshold ablation temperature (55–60°C). Therefore, we irradiated xenograft tumors using FUS at the sub-threshold ablation temperature. Sham irradiation was performed in the control group. The treatment regimen in the FUS therapy group consisted of single irradiation for one focal point. The irradiation time (Ton) of a single point was 500 ms, the irradiation interval time (Toff) was 2000 ms, the acoustic output power (P) was 47.92 W, the distance between two points was 1 mm, and the spatial average intensity was 11,340 W/cm^2^. The acoustic output power in the control group was 0 W. The tip of the thermal needle was inserted into the tumor to a depth of approximately 4 mm. The tumor temperature during the FUS therapy was monitored to ensure that the highest temperature inside the tumor was < 60°C. The treatment area was a square area of 25 mm^2^ on the X-Y plane.

MRI was performed before and 24 h, 48 h, 2 weeks after FUS treatment. Anesthetized mice were immobilized head first in a prone position in the center of the coil. The abdomen was immobilized with tape to reduce the respiratory amplitude. Mice were kept warm during imaging. The position of the mouse and height of the coil were adjusted to ensure that the center of the tumor and the center of the magnetic field were consistent. The scanning sequence included T1WI-TSE and T1WI-SPIR. The scanning parameters are shown in Table 2.

**Table 2:** MRI sequences and parameters

Parameter	T1WI-TSE	T1WI-SPIR
TR (ms)	30	500
TE (ms)	3.4	21
Field of view (mm^2^)	250 × 190	50 × 50
Slice thickness (mm)	2	2
Slice distance (mm)	0.2	0.2
Acquisition matrix	672 × 249	168 × 124

During the T1WI-SPIR sequence, 0.05 mL Gadopentetate Dimeglumine Injection Solution (Bayer, Berlin, Germany) containing 469.01 mg/mL gadopentetate dimeglumine was injected into the tail veins of nude mice followed by 1 mL of normal saline. The perfusion of the contrast agent before and after xenograft irradiation of xenografts was monitored. Mice were sacrificed 24 h, 48 h, and 2 weeks after FUS therapy (1 mouse/timepoint). Xenografts tumors were excised, fixed in 10% neutral formalin, embedded in paraffin, sectioned, stained with HE, and analyzed using TUNEL assays. The morphology and range of FUS ablation lesions in xenografts were visualized by microscopy and compared to the results of MRI and CE-MRI scans.

## CONCLUSIONS

Sub-threshold FUS induces pancreatic cancer cell apoptosis and inhibits tumor growth without damaging surrounding tissue. Lower doses induced apoptosis rather than necrosis. Therefore, only subtle differences were observed by MRI before and after FUS, while ablation lesions demonstrated non-enhanced low signal areas on CE-MRI. CE-MRI delineated the ablation lesions better 2 weeks post-treatment than in the early phase after irradiation.
